# Supplementary material for: Prevalence and Clinical Significance of Potential Drug–Drug Interactions in Hospitalized Pediatric Oncology Patients: A Prospective Pharmacoepidemiologic Study
Source: Cancers (Basel). 2025 Sep 18;17(18):3054. doi: 10.3390/cancers17183054 (PMC12469098; doi:10.3390/cancers17183054)
Supplement: Supplementary file 1 [file cancers-17-03054-s001.zip › cancers-3888484-supplementary.pdf]

Supplement

Table S1. The definition of each risk rating according to Lexi-Interact and Drugs.com

**Lexi-Interact**		
Risk	***Action***	***Description***
A	No interaction	Data have not demonstrated either pharmacodynamic or pharmacokinetic interactions between the specified agents
B	No action needed	Data demonstrate that the specified agents may interact with each other, but there is little to no evidence of clinical concern resulting from their concomitant use.
C	Monitor therapy	Data demonstrate that the specified agents may interact with each other in a clinically significant manner. The benefits of concomitant use of these two medications usually outweigh the risks. An appropriate monitoring plan should be implemented to identify potential negative effects. Dosage adjustments of one or both agents may be needed in a minority of patients.
D	Modify regimen	Data demonstrate that the two medications may interact with each other in a clinically significant manner. A patient-specific assessment must be conducted to determine whether the benefits of concomitant therapy outweigh the risks. Specific actions must be taken to realize the benefits and/or minimize the toxicity resulting from the concomitant use of the

		agents. These actions may include aggressive monitoring, empiric dosage changes, and choosing alternative agents.
X	Avoid combination	Data demonstrate that the specified agents may interact with each other in a clinically significant manner. The risks associated with concomitant use of these agents usually outweigh the benefits. These agents are generally considered contraindicated.

**Drugs.com**	
Risk	***Description***
Major	Highly clinically significant.<br>Avoid combination: The risk of the interaction outweighs the benefit.
Moderate	Moderately clinically significant.<br>Usually avoid combinations: use it only under special circumstances.
Minor	Minimally clinically significant.<br>Minimize risk: assess risk and consider an alternative drug, take steps to circumvent the interaction risk and or institute a monitoring plan.

Table S2. Comparison of severity ratings for selected drug pairs as classified by Lexi-Interact™ and Drugs.com™ interaction databases.

Drug Pair	Lexi-Interact™ Severity	Drugs.com Severity	Notes on Discrepancy
**Acetaminophen - Granisetron**	B (Moderate)	Moderate	Both indicate moderate; consistent
**Methotrexate - Vincristine**	C (Moderate)	Moderate	Both moderate; consistent
**Methotrexate - Cotrimoxazole**	D (Major)	Moderate	Lexi assigns higher severity than Drugs.com
**Pegaspargase - Vincristine**	C (Moderate)	Moderate	Consistent moderate ratings
**Ciprofloxacin - Cotrimoxazole**	C (Moderate)	Moderate	Consistent moderate ratings
**Methotrexate - Ciprofloxacin**	C (Moderate)	Moderate	Consistent moderate ratings
**Dexamethasone - Fluconazole**	C (Moderate)	Moderate	Consistent moderate ratings
**Vincristine - Fluconazole**	C (Moderate)	Moderate	Consistent moderate ratings
**Fluconazole - Granisetron**	B (Minor to Moderate)	Moderate	Lexi slightly lower severity
**Cisplatin - Fluorouracil & Leucovorin**	C (Moderate)	Major	Drugs.com gives higher severity rating
**Methotrexate - Leucovorin**	A (Minor)	Moderate	Drugs.com rates higher severity
**Ciprofloxacin - Hydrocortisone**	C (Moderate)	Moderate	Consistent moderate ratings
**Linezolid - Granisetron**	C (Moderate)	Moderate	Consistent moderate ratings
**Acetaminophen - Granisetron**	B (Moderate)	Moderate	Consistent moderate ratings
**Fluconazole - Ciprofloxacin**	B (Minor to Moderate)	Moderate	Lexi lower, Drugs.com higher
